# Adaptive gait transition in trekking pole-assisted hiking due to fatigue and staircase height elevation

**DOI:** 10.3389/fspor.2025.1669574

**Published:** 2026-01-23

**Authors:** Yusuke Koshimizu, Akira Fukuhara, Yuji Yamamoto, Akifumi Kijima

**Affiliations:** 1 Graduate School of Medicine, University of Yamanashi, Yamanashi, Japan; 2 Research Institute of Electrical Communication, Tohoku University, Miyagi, Japan; 3 Department of Psychological Sciences, Niigata University of Health and Welfare, Niigata, Japan; 4 Faculty of Education, University of Yamanashi, Yamanashi, Japan

**Keywords:** diagonality, duty factor, internal and external constraint, quadrupedal gait, symmetry, trail walking

## Abstract

Humans adapt their gait patterns in response to both internal (e.g., fatigue) and external (e.g., terrain) constraints. Although bipedal locomotion is generally efficient, its stability is reduced on steep or uneven surfaces. Under such conditions, adult hikers often use trekking poles to enhance stability and reduce physical load. In this study, we analysed pole-foot coordination in novice hikers ascending a 4.2 km uphill trail using a gait-classification framework derived from quadrupedal locomotion research. Coordination patterns were characterised by duty factor and diagonality, and gait transition was defined as a shift in the dominant diagonality range across stair-height conditions. When participants ascended moderately high stairs (20 cm), diagonal couplets (diagonality ranges of 40%–50% and 50%–60%) were most frequently observed. These patterns accounted for 36.7% of all steps recorded at 20 cm stairs in the early section of the trail and were similarly frequent (43.4%) in the later section. In contrast, when the stair height increased to approximately 40 cm, lateral couplets (diagonality ranges of 0%–10% and 90%–100%) became dominant, accounting for 33.9% of all steps recorded under this condition. These results indicate that hikers preferentially select pole-foot coordination patterns depending on stair height and show that a diagonal-lateral classification scheme provides a useful basis for describing qualitative coordination transitions in human pole-assisted gait.

## 1 Introduction

Human bipedal locomotion is generally more energy-efficient than quadrupedal gait; nonetheless, it is inherently less stable (1, 2). Consequently, infants—whose bipedal system is not yet sufficiently developed to support the body’s center-of-mass—adopt quadrupedal crawling. Even among adults with fully developed bipedal systems, locomotor stability can be substantially challenged when traversing steep inclines or uneven terrain (3–6).

Trekking poles are widely used to assist walking under such environmental conditions. Previous studies have reported that using trekking poles with both hands can improve walking efficiency relative to ordinary bipedal locomotion, while also reducing postural instability, fall risk, and overall physical load during walking (7, 8). However, despite their widespread use, the coordination patterns underlying pole-assisted locomotion have received limited systematic attention.

In this study, we aimed to characterise the orderly coordination patterns of pole-assisted gait during uphill trail walking. Rather than assuming convergence to a single stable gait pattern, we hypothesised that multiple distinct coordination patterns would emerge, with discrete transitions as a function of internal and external constraints. These transitions were not treated as gradual quantitative adjustments—such as changes in step length or stance width—but as qualitative transitions between coordination patterns, defined by the temporal organisation of limb and pole ground-contact events.

To quantify such transitions, we adopted an inter-limb coordination classification framework that was originally developed in studies on quadrupedal animal locomotion. Within this framework, gait patterns are defined by the relative timing of fore- and hind-limb ground contacts, yielding two qualitatively distinct orderly patterns: diagonal couplets, in which the contralateral fore- and hind-limbs are synchronised in-phase, and lateral couplets, in which ipsilateral fore -hind limbs are synchronised in-phase (9, 10). Because this framework is based solely on observable temporal relations among ground-contact events, it can be applied independently of the effector morphology or control architecture.

Although trekking poles lack muscular and neural systems and therefore do not operate under the same mechanical or control constraints as biological limbs, during locomotion, they function as extensions of the arms, which are coordinatively coupled with the legs. Under this functional coupling, analogous coordination patterns may emerge in pole-assisted gait. Accordingly, the present study does not assume mechanistic equivalence between quadrupedal limb coordination and human pole-assisted movement. Instead, the quadrupedal framework is strictly used as a descriptive taxonomy for classifying observable timing relations. From this perspective, we extended the timing-based classification framework to human pole-assisted locomotion by characterising pole-foot coordination using relative ground-contact timing indices, including duty factor and diagonality (see Methods for details).

The present study provides a descriptive analysis of pole-foot coordination in novice hikers walking on uneven natural mountain trails, using a timing-based classification framework extended from quadrupedal locomotion. Specifically, we examine whether diagonal and lateral couplets—coordination orders previously identified in quadrupedal animals—can be observed in human pole-assisted gait.

## 2 Material and methods

### 2.1 Participants

Overall, 20 participants, including 12 males and eight females, were recruited from students in the Department of Health and Physical Education at University of Yamanashi. All participants belonged to college athletic clubs in their respective sports. Male participants (*n* = 12) were distributed across volleyball, baseball, and track-and-field (3 each), and judo, rowing, and wrestling (1 each). Female participants (*n* = 8) played soccer (5), and volleyball, tennis, and badminton (1 each). All participants practiced as recreational or semi-competitive athletes 3–5 days per week (2–3 h per day). None of the participants had prior experience of hiking, either with or without trekking poles. Demographic characteristics, including age, height, weight, and body mass index, are summarised in Table 1. Written informed consent was obtained from all participants for their participation in the experiment, as well as for the online open-access publication of the results. This study was conducted in accordance with the principles outlined in the World Medical Association Declaration of Helsinki (2024). All participants were confirmed to be free of psychological or physiological disorders, including perceptual-motor dysfunction, based on the results of an annual health checkup at the University of Yamanashi.

**Table 1:** Demographic characteristics of participants.

Variables	Male (*N* = 12)	Female (*N* = 8)	Total (*N* = 20)
Age (years)	18.33 ± 0.471	18.000 ± 0.000	18.200 ± 0.400
Height (cm)	172.792 ± 3.322	160.538 ± 4.785	167.890 ± 7.198
Weight (kg)	68.275 ± 10.924	56.387 ± 5.312	63.520 ± 10.807
BMI (kg/$m^{2}$)	22.857 ± 3.567	21.878 ± 1.841	22.465 ± 3.036

Means ± SD for each measure.

### 2.2 Experimental task

The Participants walked along a 4.2 km hiking trail (*Yumurayama-Hachiojiyama-Tenguyama* course) for 90 min while using trekking poles in both hands. Four of the 12 staircase sections built into the trail were selected for optical recording of the participants’ gait.

The trail was primarily maintained as a hiking course, with the first half consisting mainly of staircases of nearly uniform height (≈20 cm) and depth (≈40 cm). In Contrast, the rocky section toward the end of the trail included steeper steps. The four selected staircase spots were labelled 1h-$1^{\mathrm{st}}$, 1h-$2^{\mathrm{nd}}$, 2h-$1^{\mathrm{st}}$, and 2h-$2^{\mathrm{nd}}$, with the first two located in the first half of the trail and the latter two in the second half.

The latter two spots were selected to impose greater locomotor demands. The 2h-$1^{\mathrm{st}}$ spot consisted of a rocky staircase with an unusually high step height (≈40 cm), followed by the 2h-$2^{\mathrm{nd}}$ spot with steos of usual height (≈20 cm), located near the end of the trail where fatigue had accumulated. The first two spots were chosen to ensure that the participants had adapted to pole-assisted walking but had not yet accumulated substantial fatigue. The distances between the early and late pairs of spots were matched.

**Figure 1:**
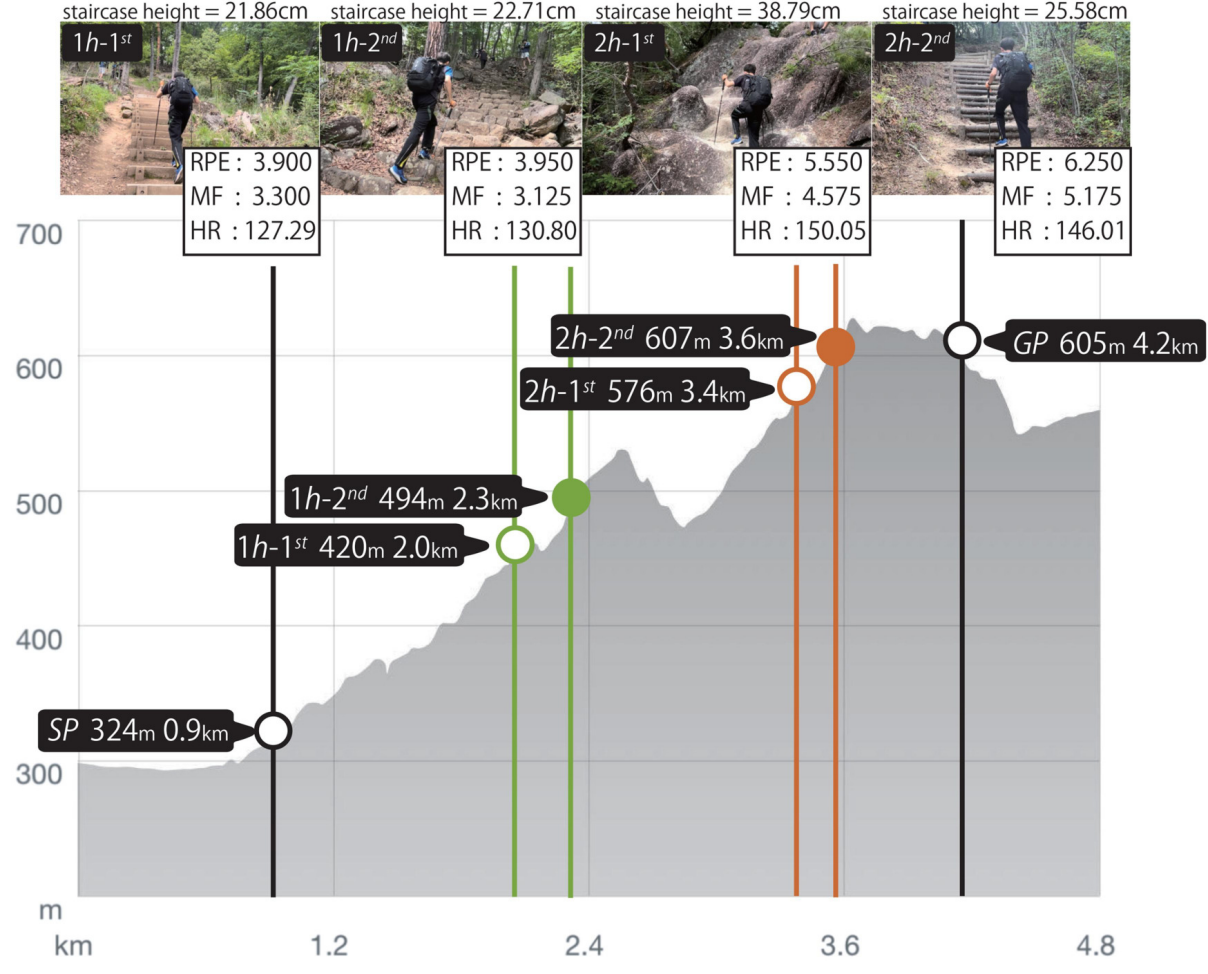
Distance and elevation profile of the hiking course, including four spots where the gait of the participants was recorded. The abscissa represents the walking distance (km), and the vertical axis indicates the elevation (m). The four recording spots (1h-$1^{\mathrm{st}}$, 1h-$2^{\mathrm{nd}}$, 2h-$1^{\mathrm{st}}$, and 2h-$2^{\mathrm{nd}}$) are marked by green and brown circles accompanied by photographs. SP and GP denote the starting and goal points, respectively. Each photograph displays the landscape of each spot, along with the stair height (cm) and average values of the rate of perceived exertion(RPE), rate of muscle fatigue(MF), and heart rate(HR).

Optical recordings were used to identify the contact and lift-off timings of four limbs: right pole tip, right foot, left pole tip, and left foot. Trekking poles were treated as functional limb extensions. Based on these timings, we calculated the duty factor, gait cycle, and diagonality. Each spot contained at least 14 stairs. Three spots had stair heights of approximately 20 cm, whereas the 2h-$1^{\mathrm{st}}$ spot had steps of approximately 40 cm. The weather conditions were clear, with slightly moist ground. The details of the hiking trail and recording spots are shown in Figure 1.

The mean and SD of the stair height, width, and depth for each spot are presented in Table 2. Although the 2h-$1^{\mathrm{st}}$ spot exhibited greater variability, SD values were not excessively large. Nevertheless, surface irregularities may have influenced gait.

**Table 2:** Size of staircases at the four measurement spots.

Variables	1h-$1^{\mathrm{st}}$	1h-$2^{\mathrm{nd}}$	2h-$1^{\mathrm{st}}$	2h-$2^{\mathrm{nd}}$
Height	21.857 ± 3.270	22.714 ± 4.333	38.786 ± 4.$739^{*}$	25.587 ± 4.315
Depth	58.786 ± 8.629	45.714 ± 9.952	42.643 ± 5.433	47.921 ± 4.752
Width	124.643 ± 6.297	148.929 ± 4.891	97.857 ± 12.$188^{*}$	122.334 ± 1.036

Mean ± SD for each measure in cm. Asterisks indicate significant difference to 1h-$1^{\mathrm{st}}$, 1h-$2^{\mathrm{nd}}$ and 2h-$2^{\mathrm{nd}}$.

### 2.3 Experimental protocol

The participants wore a heart rate (HR) monitor (Polar Unite). The trekking poles were adjusted to 68% of the participant’s height. The participants were instructed to use the poles naturally to reduce fatigue.

Participants walked 0.9 km (18 min) before reaching the trailhead to become familiar with pole-assisted locomotion and then ascended the trail. At each recording spot, the participants stopped briefly and reported ratings of perceived exertion (RPE; OMNI 0–10 scale) and muscle fatigue (MF; Borg scale 0–10). The initial fatigue levels before walking were low (HR = 108.489 ± 12.110 bpm, RPE = 1.705 ± 1.159, MF = 2.150 ± 1.492).

Two iPhone 13 cameras (30 Hz) were used to capture the staircase structure and whole-body motion. A sampling rate of 30 Hz provided sufficient temporal resolution relative to the typical stair-climbing gait frequency (∼2 Hz), allowing reliable detection of ground-contact timing. Approximately seven gait cycles (two steps per cycle) were recorded at each spot.

After completing the recordings at the four spots, the participants continued to the summit and then descended to the goal. All protocols were approved by the Research Ethics Committee of the Faculty of Education, University of Yamanashi.

### 2.4 Data analysis

#### 2.4.1 Fatigue estimation

Fatigue was assessed using HR and psychological measures (RPE and MF). A 10 s HR segment recorded at each spot was used as a physiological index. The RPE and MF reported before each ascent were used as psychological indices. Blood lactate was not measured because of field constraints; therefore, all fatigue comparisons were relative, focusing on differences between the early and late phases of the trail.

#### 2.4.2 Calculation of duty factor and diagonality

The duty factor was defined as the proportion of the gait cycle during which each limb remained in ground contact. The diagonality was defined as the time lag between the left-foot and left-pole contact. Both were normalized to % gait cycle. Contact and lift-off timings were determined by frame-by-frame inspection of a 30 Hz video using iMovie. To address concerns regarding temporal resolution and sampling density, the 30 Hz frame rate (±33 ms uncertainty) was evaluated relative to the stair-climbing gait dynamics. Given a typical gait frequency of ∼2 Hz and stance durations of at least 150–200 ms, the timing uncertainty was less than 7% of the stance phase and substantially smaller than the 10%-width diagonality bins used for classification. Thus, frame-resolution limitations are unlikely to cause bin misclassification.

Although only seven gait cycles were recorded at each spot, diagonality is a phase-based measure of relative limb timing and is known to stabilise rapidly. Prior studies of human locomotor coordination show that phase relationships typically reach steady values within single cycles across gait transitions (11–13). Thus, seven cycles provided a sufficient window to capture stable coordination. Furthermore, diagonality was evaluated as a frequency distribution within predefined bins (10 for sequence, five for couplets), following standard analytical practice in quadrupedal and primate locomotion, in which the limb phase is routinely assessed using binned or histogram-based representations (14–17). This bin-based approach reduces cycle-to-cycle variability and yields robust pattern estimation, even with modest cycle counts. Therefore, the number of gait cycles available at each spot was adequate for characterising stable coordination patterns.

It is important to emphasise that trekking poles were not treated as biomechanical limbs in a morphological sense, but rather as functional contact elements contributing to the support polygon during ascent. Accordingly, duty factor and diagonality were not interpreted using biological limb-swing semantics, but strictly as phase-based descriptors of the temporal organisation of contact events. This abstraction is consistent with the use of Hildebrand-style phase classifications across taxa and mechanical systems, in which “limbs” denote any repeated contact sequence rather than a homologous anatomy. Thus, applying this framework to pole-foot coordination addresses coordination geometry, rather than limb biology.

#### 2.4.3 Gait pattern estimation using a nonlinear function of diagonality

To estimate the coordination patterns, the diagonality function was parameterised using the duty factors of the poles and feet. The duty factor and gait-cycle values were computed as the mean across seven gait cycles at each spot for each participant. In accordance with the a priori definition of diagonality, cycles in which a pole did not contact the ground before the subsequent left-foot contact (i.e., pole duty factor ≈0%) were excluded because the pole-foot phase lag was undefined in such cases. These instances constituted only 2.7% (15 cycles) of all recorded data and were distributed across participants and spots, indicating that their exclusion did not bias the distribution.

Statistical analysis confirmed that pole duty factors were significantly lower than foot duty factors (F(3,57)=60.270, p<0.001, $\eta_{p}^{2}=0.760$), as shown in Table 3. Across all spots, the pole duty factors remained slightly below 50%, whereas the foot duty factors were higher. Therefore, representative values of 48.3% for poles and 59.3% for feet—each averaged across limbs and spots—were used in subsequent analyses.

**Table 3:** Duty factor for each of the four limbs across the four spots. For each participant, the mean value across seven gait cycles at each spot was calculated, and these means were then averaged across all 20 participants.

Limb	Left/Right	1h-$1^{\mathrm{st}}$	1h-$2^{\mathrm{nd}}$	2h-$1^{\mathrm{st}}$	2h-$2^{\mathrm{nd}}$	Mean ± S.D.
Poles	Right	47.1 ± 8.5	48.6 ± 7.6	46.0 ± 9.4	49.4 ± 11.8	47.8 ± 1.5
	Left	49.1 ± 10.2	49.0 ± 7.0	48.2 ± 9.5	49.1 ± 13.4	48.8 ± 0.4
${\mathrm{Feet}}^{*}$	Right	58.9 ± 2.0	59.3 ± 4.4	60.9 ± 3.0	59.1 ± 2.9	59.5 ± 0.9
	Left	58.7 ± 2.5	60.1 ± 3.3	60.3 ± 3.0	58.0 ± 2.6	59.3 ± 1.2

The mean ± S.D. indicates those among the four spots. An asterisk (*) indicates a value significantly greater than that for the poles.

**Figure 2:**
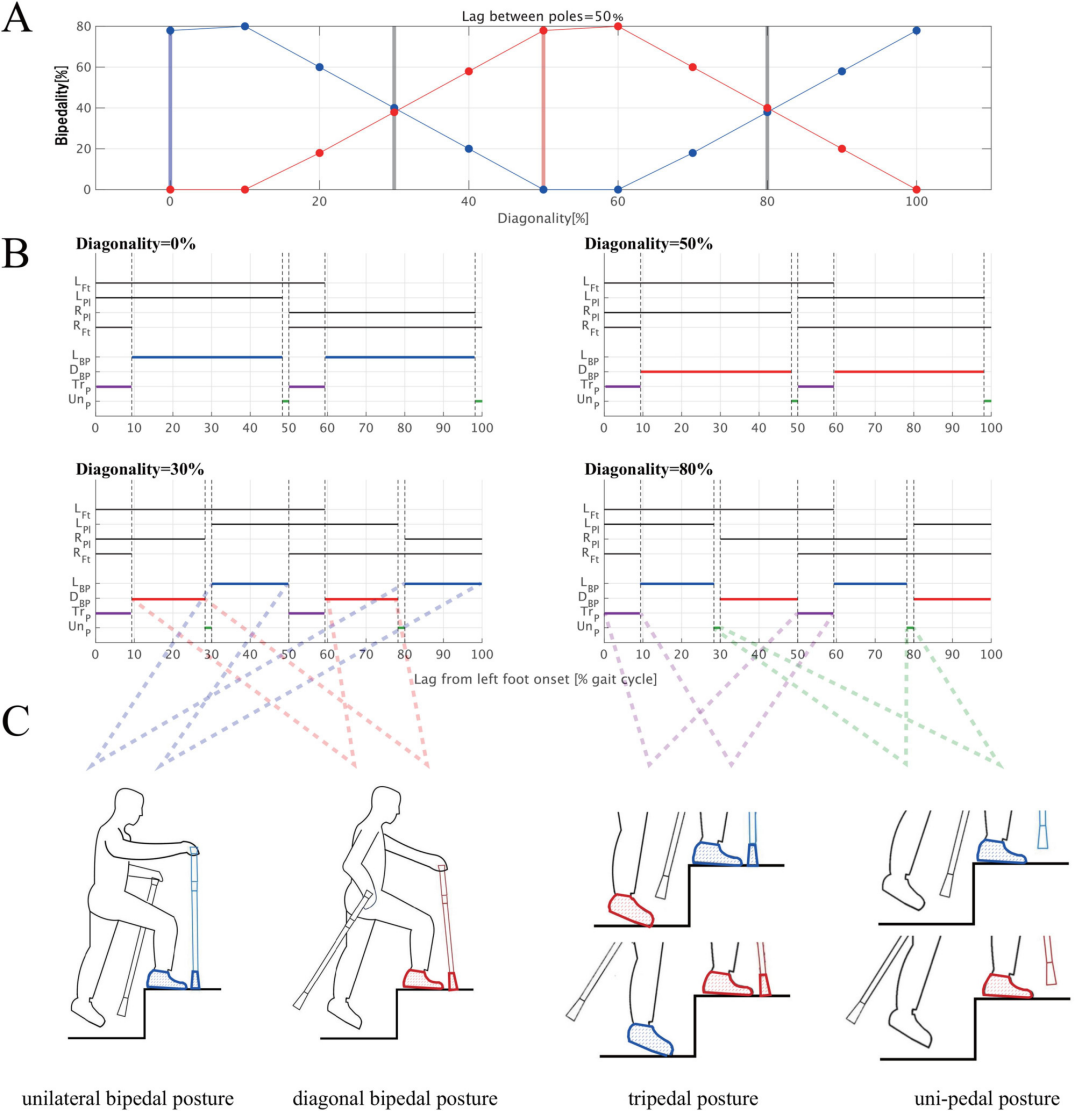
Probability of the bipedality emergence as a function of diagonality. **(A)** Percentage of unilateral (blue solid line) and diagonal (red solid line) bipedality relative to a gait cycle. The blue vertical line indicates the onset of a phase dominated by unilateral bipedality, whereas the red vertical line indicates the onset of a phase dominated by diagonal bipedality. The gray vertical lines indicate the crossing points at which the frequencies of the two bipedal gait patterns were reversed. The data are from a typical patient trekking trial. **(B)** Each of the top four black solid lines in each panel indicates the times when each of four limbs were in contact with the ground ($L_{\mathrm{Ft}}$: left foot, $L_{\mathrm{Pl}}$: left pole, $R_{\mathrm{Pl}}$: right pole, $R_{\mathrm{Ft}}$: right foot), and each of lower four coloured lines indicate unilateral bipedal phase ($L_{\mathrm{BP}}$: blue), diagonal bipedal phase ($D_{\mathrm{BP}}$: red), tripedal phase (${\mathrm{Tr}}_{P}$: purple) and uni-pedal phase (${\mathrm{Un}}_{P}$: green). Tripedal refers to periods when three of the four limbs are in contact, and uni-pedal refers to the periods when only one limb is in contact. **(C)** Postures of each gait. Top: unilateral (left) and diagonal (right) bipedality. Bottom: Tripedal postures (left) and uni-pedal posture (right).

Figure 2A illustrates the transition between lateral and diagonal bipedality as a function of diagonality. Panel A depicts changes in the duration of unilateral bipedality—identified by simultaneous ground contact of the ipsilateral pole-foot pair—and diagonal bipedality—characterised by simultaneous contact of the contralateral pole-foot pair—as functions of diagonality. The durations of unilateral and diagonal bipedality peaked when diagonality ranged from 0% (or 100%) to 10% and from 50% to 60%,respectively. In these ranges, an alternative pattern did not occur. This mutual exclusivity is further illustrated in the two upper panels labelled “Diagonality = 0%” and “Diagonality = 50%” in Figure 2B. Regardless of diagonality, the left and right feet alternately contacted the ground with a lag of approximately 50% of the gait cycle, including two phases of simultaneous contact (ranging from 0%–9.3% to 50.0%–59.3% across panels). Furthermore, Figure 2B shows the uni-pedal and tripedal phases. The uni-pedal and tripedal phases indicate the periods during which only one foot and three limbs—both feet and either the left or right pole—, respectively, are in contact with the ground. Quadrupedal phases, in which all four limbs simultaneously contacted the ground, were not observed because the participants were prohibited from stopping at any of the four measurement spots.

To ensure that this analysis was applicable to our data, we examined the timing differences between the left and right poles and assessed their impact on bipedality estimation before performing the estimation itself. Although prior studies (9) that proposed the estimation model shown in Figure 2 considered asymmetry in the duty factor between the fore and hind limbs, they did not explicitly address left-right ground contact timing delays in the fore and hind limbs. Specifically, they typically assumed that the fore and hind limbs alternate with an approximate half-cycle phase lag. Therefore, we verified the validity of this assumption for pole-foot coordination gaits and determined how left-right asymmetry influences estimation accuracy.

Having ensured the validity of the analytical method, we confirmed gait characteristics using two variables—*sequence* and *couplets*—based on the diagonality magnitude. First, consistent with the definition of diagonality, we confirmed the ipsilateral pole-foot *sequence* by calculating the pole contact delay relative to foot contact. In this analysis, gaits with diagonality of 0% and 100% have distinct implications: the former indicates immediate pole landing following ipsilateral foot contact, whereas the latter signifies substantially delayed pole contact, approaching a complete gait cycle. Therefore, to examine the *sequence*, we created a histogram using 10 bins (0%–10%, 10%–20%, …, 90%–100%) to identify directional bias in the distribution of diagonality values across strides. In contrast, when evaluating pole-foot *couplets* solely based on the temporal proximity of ground contact, regardless of contact order, diagonality values of 0% and 100% are functionally equivalent. Here, a diagonality of 50% represents simultaneous contact of the contralateral pole and foot, and diagonality pairs equidistant from 50% (e.g., 10% and 90%, 20% and 80%) reflect similar couplets. Therefore, to evaluate gait *couplets*, we grouped stride frequencies within the following symmetric ranges: [0–10]+[90–100]%, [10–20]+[80–90]%, [20–30]+[70–80]%, [30–40]+[60–70]%, and [40–50]+[50–60]%. For each participant, we calculated the frequencies for each of the 10 or five bins.

#### 2.4.4 Statistical procedure

A one-way repeated-measures ANOVA was applied to HR, RPE, and MF to test for differences across the four spots (1h-1^st^, 1h-2^nd^, 2h-1^st^, and 2h-2^nd^).

To examine whether the left-right pole lag and inter-foot lag deviated systematically from the 50% reference value, we conducted a three-way repeated-measures ANOVA with factors of limb (2: feet, poles), spot (4 levels), and lag region (10 levels: 0%–10%, …, 90%–100%).

After validating the diagonality model, the gait-pattern frequencies were analysed using either 10 bins (sequence) or five symmetric bins (couplets). For the three spots with equal stair heights, a two-way repeated-measures ANOVA (spot × diagonality region) was applied. A separate two-way ANOVA was used to compare the stair-height effects between 2h-1^st^ and 2h-2^nd^. Violations of sphericity were corrected using the Greenhouse-Geisser adjustment. Effect sizes were reported uniformly as $\eta_{p}^{2}$. Significant interactions were followed by simple main effect analyses with Bonferroni correction for multiple comparisons.

## 3 Results

### 3.1 Psychological and physiological fatigue

The results of the one-way repeated-measures ANOVA revealed a significant effect of the trail spot on all three fatigue measures, as shown in Table 4.

**Table 4:** Increase in psychological and physiological fatigue across different hiking trail spots. The values of psychological fatigue included rate of perceived exertion (RPE), muscle fatigue (MF), and heart rate (HR). SDs represent the standard error across 20 participants.

Measure	1h-$1^{\mathrm{st}}$	1h-$2^{\mathrm{nd}}$	2h-$1^{\mathrm{st}}$	2h-$2^{\mathrm{nd}}$
RPE	3.900 ± 1.758	3.950 ± 2.037	5.550 ± 2.$418^{*}$	6.250 ± 2.$364^{*}$
MF	3.300 ± 2.261	3.125 ± 2.376	4.575 ± 2.$614^{*}$	5.175 ± 2.$466^{*}$
HR	127.298 ± 16.181	130.804 ± 16.744	150.053 ± 19.$095^{*}$	146.016 ± 14.$229^{*}$

Asterisks indicate significantly higher values compared to 1h-$1^{\mathrm{st}}$ and 1h-$2^{\mathrm{nd}}$ (p<.05).

For RPE, Mauchly’s test of sphericity indicated a violation of the sphericity assumption (Mauchly’s W = 0.685, *p* = 0.024); therefore, the Greenhouse-Geisser correction was applied. The main effect of trail spot was significant (F(1.508, 28.654) = 19.304, *p* = 0.001, $\eta_{p}^{2}$ = 0.504, 1−β = 1.000). Post hoc Bonferroni-adjusted comparisons demonstrated that RPE scores at both 2h-$1^{\mathrm{st}}$ (M = 4.575) and 2h-$2^{\mathrm{nd}}$ (M = 5.175) were greater than those at 1h-$1^{\mathrm{st}}$ (M = 3.300). The difference for 2h-$1^{\mathrm{st}}$ was marginally significant (t(19) = 2.935, adjusted *p* = 0.050), whereas that for 2h-$2^{\mathrm{nd}}$ was statistically significant (t(19) = 4.434, adjusted *p* = 0.002). No significant differences were observed between 1h-$1^{\mathrm{st}}$ and 1h-$2^{\mathrm{nd}}$, or between 2h-$1^{\mathrm{st}}$ and 2h-$2^{\mathrm{nd}}$, indicating that RPE scores remained stable within each half of the trail.

For MF, Mauchly’s test of sphericity indicated a violation of the sphericity assumption (Mauchly’s W = 0.614, *p* = 0.007); therefore, the Greenhouse-Geisser correction was applied. The main effect of trail spot was significant (F(1.978, 37.574) = 41.674, *p* = 0.001, $\eta_{p}^{2}$ = 0.687, 1−β = 1.000). Post hoc Bonferroni-adjusted comparisons indicated that MF scores at both 2h-$1^{\mathrm{st}}$ (M = 5.550) and 2h-$2^{\mathrm{nd}}$ (M = 6.250) were significantly higher than those at 1h-$1^{\mathrm{st}}$ (M = 3.900; vs. 2h-$1^{\mathrm{st}}$: t(19) = 5.051, adjusted *p* = 0.001; vs. 2h-$2^{\mathrm{nd}}$: t(19) = 7.194, adjusted *p* = 0.001) and at 1h-$2^{\mathrm{nd}}$ (M = 3.950; vs. 2h-$1^{\mathrm{st}}$: t(19) = 8.107, adjusted *p* = 0.001; vs. 2h-$2^{\mathrm{nd}}$: t(19) = 9.516, adjusted *p* = 0.001). No significant differences were observed between 1h-$1^{\mathrm{st}}$ and 1h-$2^{\mathrm{nd}}$, or between 2h-$1^{\mathrm{st}}$ and 2h-$2^{\mathrm{nd}}$.

The main effect of trail spot was also significant for HR. Mauchly’s test of sphericity indicated a violation of the sphericity assumption (Mauchly’s W = 0.425, *p* = 0.010); accordingly, the Greenhouse-Geisser correction was applied. The corrected analysis confirmed that the main effect of trail spot remained significant (F(1.900, 36.098) = 29.220, *p* = 0.001, $\eta_{p}^{2}$ = 0.606, 1−β = 1.000). Post hoc Bonferroni-adjusted comparisons demonstrated that HR scores at both 2h-$1^{\mathrm{st}}$ (M = 150.050) and 2h-$2^{\mathrm{nd}}$ (M = 146.020) were significantly higher than those at 1h-$1^{\mathrm{st}}$ (M = 127.300; vs. 2h-$1^{\mathrm{st}}$: t(19) = 5.456, adjusted *p* = 0.001; vs. 2h-$2^{\mathrm{nd}}$: t(19) = 6.446, adjusted *p* = 0.001) and 1h-$2^{\mathrm{nd}}$ (M = 130.800; vs. 2h-$1^{\mathrm{st}}$: t(19) = 6.736, adjusted *p* = 0.001; vs. 2h-$2^{\mathrm{nd}}$: t(19) = 7.367, adjusted *p* = 0.001). No significant differences were observed between 1h-$1^{\mathrm{st}}$ and 1h-$2^{\mathrm{nd}}$, or between 2h-$1^{\mathrm{st}}$ and 2h-$2^{\mathrm{nd}}$. These results indicate that continuous walking on a 1.6 km uphill trail significantly increases psychological and physiological fatigue.

### 3.2 The effect of the asymmetry in left-right poles movement on the bipedality estimation

**Figure 3:**
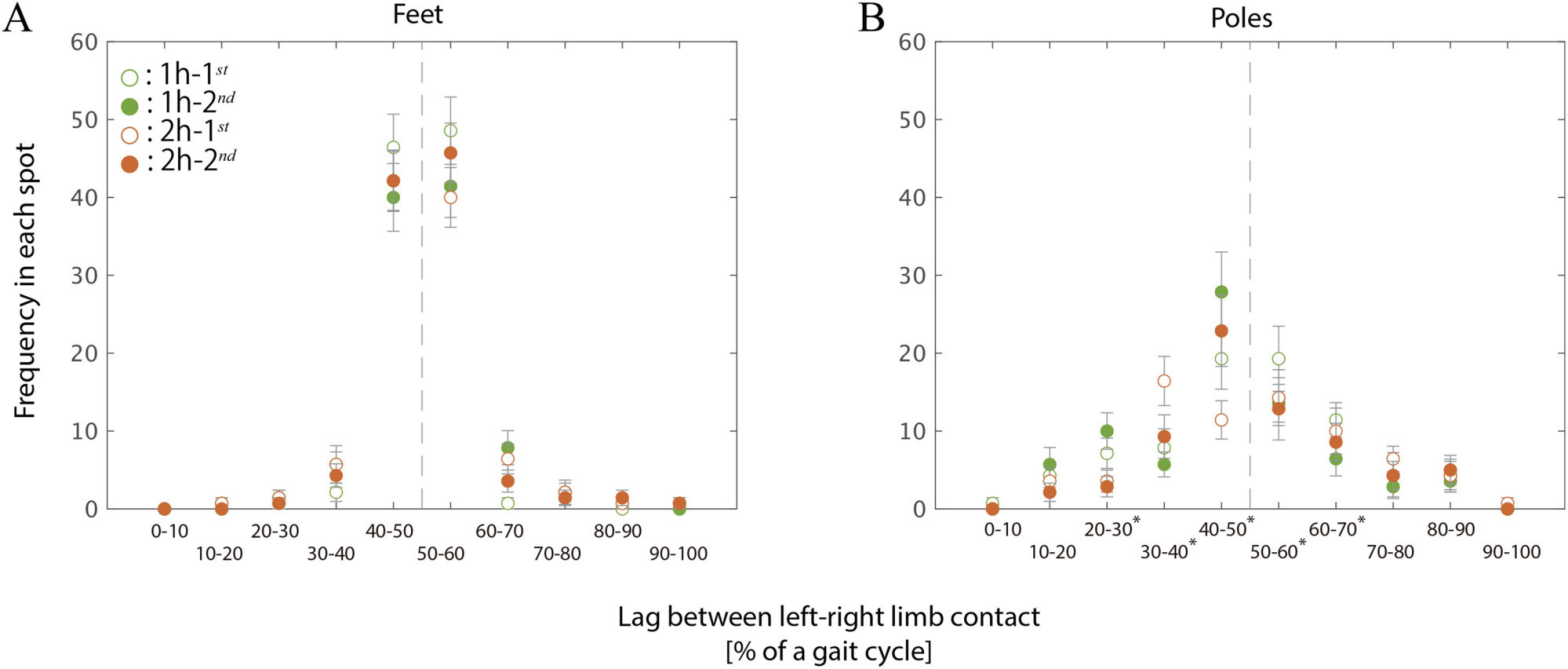
Distribution of the lag between left-right limb contacts. The left **(A)** and right **(B)** panels indicate the frequency of occurrence for the feet and poles, respectively. The representative value of the lag for each of the 20 participants was calculated as the mean of seven gait cycles measured at each of the four spots. Each of the four histograms is for each spot (blank green: 1h-$1^{\mathrm{st}}$, filled green: 1h-$2^{\mathrm{nd}}$, blank brown: 2h-$1^{\mathrm{st}}$, filled brown: 2h-$2^{\mathrm{nd}}$).

The analysis of the left-right foot lag, shown in Figure 3A, supports the validity of our analytical approach. Similarly, the trekking poles alternated in the ground contact, as evidenced by the right panel of Figure 3B. Nonetheless, the pole-pole lag was broadly distributed from 30% to 70%, although it was centered at approximately around 50%.

Three-way ANOVA with repeated measures revealed a significant main effect of limbs (F(1,19) = 58.243, p<0.001, $\eta_{p}^{2}$ = 0.754) and lag region (F(3.033, 57.080) = 120.618, p<0.001, $\eta_{p}^{2}$ = 0.864) as well as a significant interaction between limbs and lag region (F(3.033,57.612) = 54.210, p<0.001, $\eta_{p}^{2}$ = 0.741). Analysis of simple main effects of the limb on each of the 10 lag regions revealed significant effects for five regions spanning from 20%–30% to 60%–70% (20%–30%: F(1,19) = 18.870, *p* = 0.004, $\eta_{p}^{2}$ = 0.498; 30%–40%: F(1,19) = 23.297, *p* = 0.001, $\eta_{p}^{2}$ = 0.551; 40%–50%: F(1,19) = 404.462, p<0.001, $\eta_{p}^{2}$ = 0.955; 50%–60%: F(1,19) = 679.341, p<0.001, $\eta_{p}^{2}$ = 0.973; 60%–70%: F(1,19) = 16.179, *p* = 0.009, $\eta_{p}^{2}$ = 0.460). According to Figure 3B, between-pole lag tended to distribute not only around 50% of the gait cycle but also within relatively shorter regions around 20%–40% and longer regions around 60%–70%, in contrast with the foot pattern displayed in the left panel.

As shown in our calculations in Supplementary Figure S1, the magnitude of between-pole lag in the 20%–40% or 60%–70% regions did, in fact, influence the respective probabilities of unilateral and diagonal bipedality, which varied depending on diagonality. Nonetheless, even when the lag between poles varied from 10% to 90%, the frequencies of unilateral and diagonal bipedality continued to fluctuate symmetrically and in opposite phases. Therefore, to enhance the reliability of our estimation, 0.357% of the measured data in which the pole-pole lag fell outside this range was excluded. In the transition ranges—typically illustrated in the bottom two panels of Figure 2B—both bipedality patterns co-occur, and the dominant pattern transitions at diagonality values around 30% and 80%. Although these transition points vary slightly depending on the pole-pole lag shown in Supplementary Figure S1, the overall correspondence between diagonality regions and the dominant coordination pattern was maintained until the lag decreased below 20% of the gait cycle.

### 3.3 The dominant gait pattern at each trail spot

#### 3.3.1 Sequence

##### 3.3.1.1 The effects of fatigue

The two-way repeated-measures ANOVA showed that the main effect of trail spot was not significant (F(2, 38) = 1.381, *p* = 0.263, $\eta_{p}^{2}$ = 0.067, 1−β = 0.496). Mauchly’s test of sphericity indicated violations for both the main effect of the diagonality region (Mauchly’s W = 0.001, p<0.001) and the trail spot × diagonality region interaction (W = 0, *p* = 0.015). Therefore, the Greenhouse-Geisser correction was applied. After correction, the main effect of the diagonality region was significant (F(4.493, 85.363) = 4.507, *p* = 0.002, $\eta_{p}^{2}$ = 0.192, 1−β = 0.998), whereas the interaction effect did not reach statistical significance (F(8.233, 156.431) = 1.600, *p* = 0.126, $\eta_{p}^{2}$ = 0.078, 1−β = 0.954). Bonferroni-adjusted post hoc comparisons revealed that the frequency in the 40%–50% region was significantly higher than those in the 20%–30% (t(59) = 3.569, *p* = 0.032), 70%–80% (t(59) = 4.031, *p* = 0.007), and 80%–90% (t(59) = 4.144, *p* = 0.005) regions.

These findings indicate that pole contact typically lagged ipsilateral foot contact by approximately 50% of the gait cycle, resulting in diagonal pole-foot synchronisation. At all 20-cm staircase spots, foot contact slightly preceded the diagonally paired pole contact (see the 40%–50% region at the 1h-$1^{\mathrm{st}}$, 1h-$2^{\mathrm{nd}}$, and 2h-$2^{\mathrm{nd}}$ spots in Figure 5).

##### 3.3.1.2 The effects of staircase height

The results of the two-way repeated-measures ANOVA revealed that the main effect of trail spot was not significant (F(1, 19) = 1.305, *p* = 0.267, $\eta_{p}^{2}$ = 0.064, 1−β = 0.374). Mauchly’s test of sphericity indicated a violation of the main effect of the diagonality region (Mauchly’s W = 0.012, *p* = 0.009). Therefore, the Greenhouse-Geisser correction was applied. After correction, the main effect of the diagonality region was significant (F(4.920, 93.486) = 3.463, *p* = 0.007, $\eta_{p}^{2}$ = 0.153, 1−β = 0.995). Additionally, the interaction between trail spot and diagonality region was significant (F(9, 171) = 3.982, p<0.001, $\eta_{p}^{2}$ = 0.173, 1−β = 0.998).

**Figure 4:**
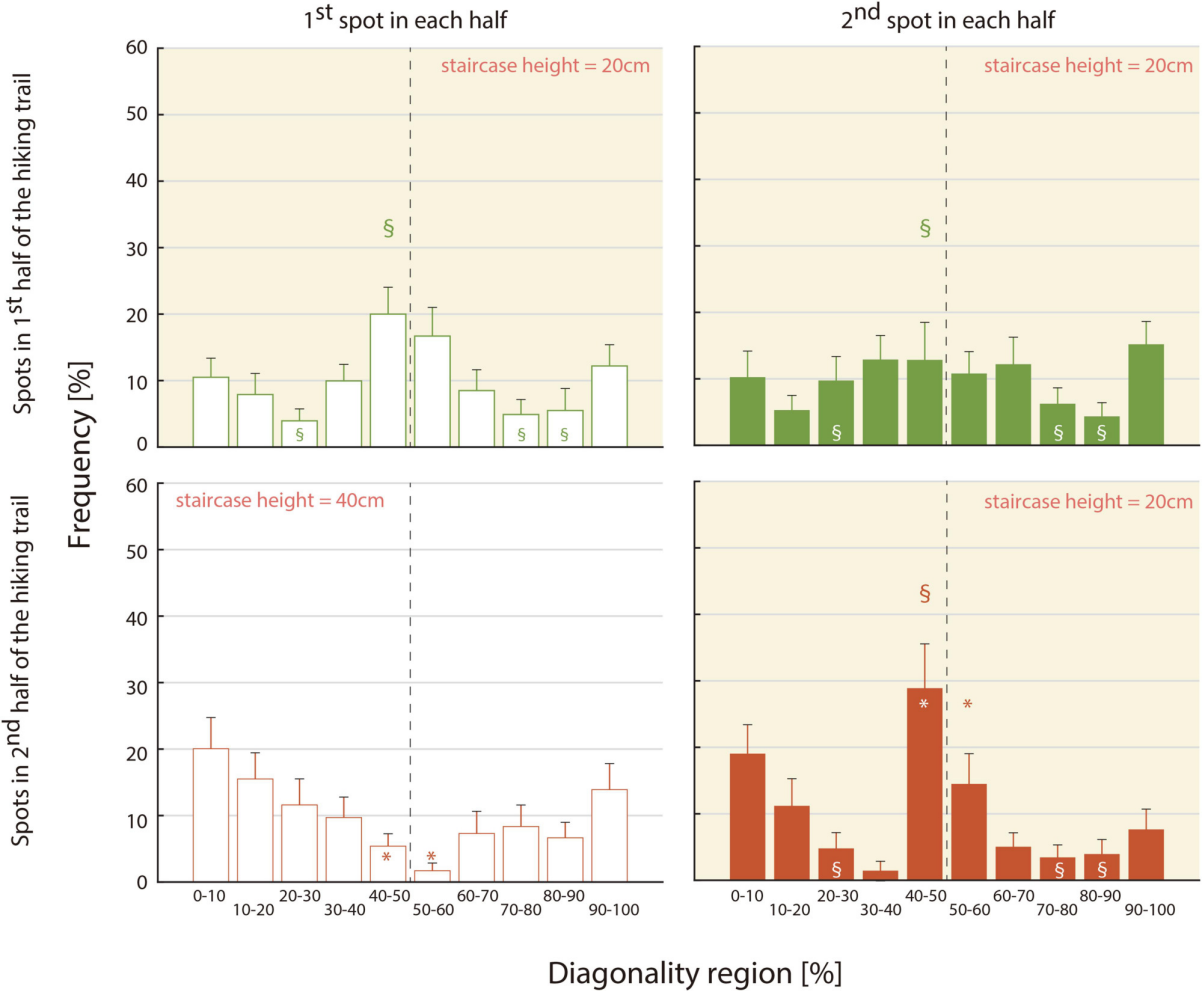
Each histogram depicts diagonality distributions measured at one of four spots. The 10 bins per histogram represent diagonality regions spanning 0% to 100% of the gait cycle. Error bars represent the standard error among 20 participants. §: Significant main effect of the diagonality regions in three spots with 20 cm staircase height (i.e., 1h-$1^{\mathrm{st}}$, 1h-$2^{\mathrm{nd}}$, and 2h-$2^{\mathrm{nd}}$). ∗: Significant simple main effect of trail spots with different staircase height (20 vs. 40 cm, i.e., 2h-$1^{\mathrm{st}}$ and 2h-$2^{\mathrm{nd}}$).

Simple main effects analysis (α = 0.15) revealed a significant simple main effect of the trail spot in the 40%–50% (F(1, 19) = 11.912, adjusted *p* = 0.016) and 50%–60% (F(1, 19) = 8.012, adjusted *p* = 0.043) regions. As shown in Figure 4, the frequencies at 2h-$1^{\mathrm{st}}$ (5.4% for 40%–50%, 1.7% for 50%–60%) were significantly lower than those at 2h-$2^{\mathrm{nd}}$ (28.8% for 40%–50%, 14.5% for 50%–60%). In addition, the simple main effect of the diagonality region was also significant at the 2h-$1^{\mathrm{st}}$ spot (F(9, 171) = 2.256, adjusted *p* = 0.021, $\eta_{p}^{2}$ = 0.106); however, Bonferroni-adjusted post hoc comparisons did not show any significant differences between regions.

The simple main effect of the diagonality region was also significant also at the 2h-$2^{\mathrm{nd}}$ spot (F(9, 171) = 4.897, adjusted p<0.001, $\eta_{p}^{2}$ = 0.206). Post-hoc comparisons showed that the frequency in the 40%–50% region (28.8%) was significantly greater than those in the 20%–30% (4.8%; t(19) = 3.066, adjusted *p* = 0.041), 30%–40% (1.4%; t(19) = 3.931, adjusted *p* = 0.035), 60%–70% (5.0%; t(19) = 3.161, adjusted *p* = 0.039), 70%–80% (3.4%; t(19) = 3.447, adjusted *p* = 0.039), and 80%–90% (3.9%; t(19) = 3.339, adjusted *p* = 0.039) regions. Additionally, the comparison between the 40%–50% and 90%–100% regions did not reach statistical significance after adjustment (t(19) = 2.496, adjusted *p* = 0.082).

These results indicate that the frequencies in the 40%–60% region at the 2h-$1^{\mathrm{st}}$ spot were lower than those at the 2h-$2^{\mathrm{nd}}$ spot. In contrast, at the 2h-$2^{\mathrm{nd}}$ spot, the distribution was characterised by a concentration of frequencies in the 40%–60% diagonality region relative to the 2h-$2^{\mathrm{nd}}$ spot.

#### 3.3.2 Couplets

##### 3.3.2.1 The effect of fatigue

The results of the two-way repeated-measures ANOVA revealed that the main effect of trail spot was not significant (F(2, 38) = 0.169, *p* = 0.845, $\eta_{p}^{2}$ = 0.009, 1−β = 0.092). Mauchly’s test of sphericity indicated a violation of the assumption for the main effect of the diagonality region (Mauchly’s W = 0.223, *p* = 0.002). Therefore, the Greenhouse-Geisser correction was applied. After applying the correction, the main effect of the diagonality region was significant (F(2.396, 44.908) = 8.712, adjusted *p* = 0.001, $\eta_{p}^{2}$ = 0.314, 1-β = 1.000).

Post-hoc comparisons using the Bonferroni correction revealed that frequencies were significantly higher in the [40–50]+[50–60]% diagonality region (34.6%) than in the [10–20]+[80–90]% (12.7%; t(59) = 4.022, adjusted *p* = 0.002), [20–30]+[70–80]% (11.0%; t(59) = 4.676, adjusted *p* = 0.001), and [30–40]+[60–70]% (16.7%; t(59) = 3.614, adjusted *p* = 0.006) regions. Additionally, the frequencies in the [0–10]+[90–100]% region (24.9%) were significantly higher than those in the [20–30]+[70–80]% region (t(59) = 3.341, adjusted *p* = 0.015).

**Figure 5:**
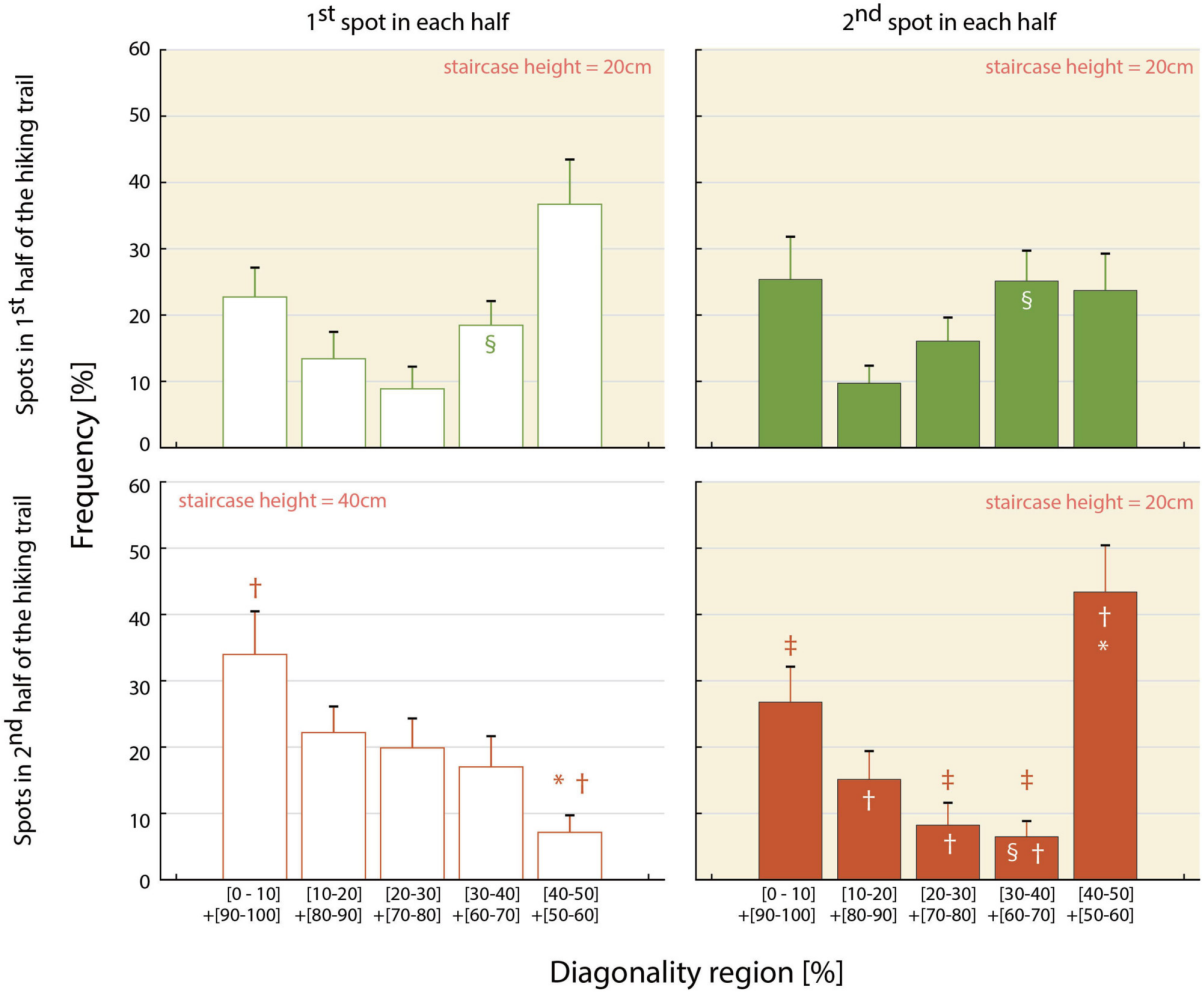
Each of the four histograms shows the distributions of diagonality measured in each of the four spots. Each histogram contains five regions representing the diagonality ranges: [0–10]+[90–100]%, [10–20]+[80–90]% … [40–50]+[50–60]% of the gait cycle. Error bars represent standard error for the 20 participants. §: significant main effect of the diagonality regions in three spots with equally low staircase height, including 1h-$1^{\mathrm{st}}$, 1h-$2^{\mathrm{nd}}$, and 2h-$2^{\mathrm{nd}}$. ∗: significant simple main effect of trail spots with different staircase height (20 vs. 40 cm), both in the late half of the trail, including 2h-$1^{\mathrm{st}}$ and 2h-$2^{\mathrm{nd}}$. † and ‡: significant simple main effect of diagonality regions in each of the two trail spots in the late half of the trail (2h-$1^{\mathrm{st}}$ and 2h-$2^{\mathrm{nd}}$).

In contrast, the interaction between trail spot and the diagonality region narrowly missed the level of statistical significance (F(8, 152) = 1.996, *p* = 0.054, $\eta_{p}^{2}$ = 0.095, 1-β = 0.904). For reference, a simple main effects analysis (with α = 0.15) revealed a significant effect of trail spot in the [30–40]+[60–70]% diagonality region (F(2, 38) = 5.858, adjusted *p* = 0.016). As shown in Figure 5, frequencies at 2h-$2^{\mathrm{nd}}$ (6.5% for [30–40]+[60–70]%) were significantly lower than those at 1h-$1^{\mathrm{st}}$ (18.4% for [30–40]+[60–70]%) and 1h-$2^{\mathrm{nd}}$ (25.1% for [30–40]+[60–70]%). Furthermore, the simple main effect of the diagonality region was also significant at the 2h-$2^{\mathrm{nd}}$ spot (F(4, 76) = 7.853, adjusted p<0.001, $\eta_{p}^{2}$ = 0.292). Post-hoc comparisons demonstrated that the frequency in the [0–10]+[90–100]% region (26.8%) was significantly greater than that in the [20–30]+[70–80]% (8.2%; t(19) = 2.518, adjusted *p* = 0.042) and [30–40]+[60–70]% (6.5%; t(19) = 3.182, adjusted *p* = 0.016) regions. Additionally, the frequency in the [40–50]+[50–60]% region (43.4%) was significantly higher than those in the [10–20]+[80–90]% (15.1%; t(19) = 2.678, adjusted *p* = 0.037), [20–30]+[70–80]% (t(19) = 4.050, adjusted *p* = 0.003), [30–40]+[60–70]% (t(19) = 4.764, adjusted *p* = 0.001) regions.

These results indicate that, under the 20 cm staircase condition, the frequency of diagonal couplets corresponding to the 40%–60% diagonality region was relatively high.

##### 3.3.2.2 The effect of staircase height

The results of the two-way repeated-measures ANOVA revealed that the main effect of trail spot was not significant (F(1,19) = 0.322, *p* = 0.577, $\eta_{p}^{2}$ = 0.017, 1−β = 0.131). Mauchly’s test of sphericity indicated a violation of the assumption for the main effect of the diagonality region (Mauchly’s W = 0.195, *p* = 0.001). Therefore, the Greenhouse-Geisser correction was applied. After correction, the main effect of the diagonality region was significant (F(2.418, 45.942) = 3.799, *p* = 0.023, $\eta_{p}^{2}$ = 0.167, 1−β = 0.873). Moreover, the interaction between trail spot and the diagonality region was significant (F(4, 76) = 7.861, p<0.001, $\eta_{p}^{2}$ = 0.293, 1−β = 0.999).

A simple main effects analysis (α = 0.15) revealed a significant simple main effect of trail spot in the [40–50]+[50–60]% region (F(1, 19) = 23.797, p<0.001). As shown in Figure 5, frequencies at 2h-$1^{\mathrm{st}}$ (7.1%) were significantly lower than those at 2h-$2^{\mathrm{nd}}$ (43.4%). A simple main effect of the diagonality region was also significant at the 2h-$1^{\mathrm{st}}$ spot (F(4, 76) = 4.019, *p* = 0.015, $\eta_{p}^{2}$ = 0.149). Post-hoc comparisons demonstrated that the frequency in the [0–10]+[90–100]% region (33.9%) was significantly higher than that in the [40–50]+[50–60]% (7.1%; t(19) = 3.165, adjusted *p* = 0.005) regions. The simple main effect of the diagonality region was significant also at the 2h-$2^{\mathrm{nd}}$ spot (F(4, 76) = 7.853, p<0.001, $\eta_{p}^{2}$ = 0.292). Post hoc comparisons showed that the frequency in the [0–10]+[90–100]% region (26.8%) was significantly greater than those in the [20–30]+[70–80]% (8.2%; t(19) = 2.518, adjusted *p* = 0.042), [30–40]+[60–70]% (6.5%; t(19) = 3.182, adjusted *p* = 0.016). Additionally, the frequency in the [40–50]+[50–60]% region (43.4%) was significantly greater than those in the [10–20]+[80–90]% region (15.1%; t(19) = 2.678, adjusted *p* = 0.037), [20–30]+[70–80]% (t(19) = 4.050, adjusted *p* = 0.003), [30–40]+[60–70]% (t(19) = 4.764, adjusted *p* = 0.001).

The results indicate that the gait pattern emergence was influenced by the height of the stair-like trail segments. Specifically, frequencies in the [40–50]+[50–60]% diagonality region—typically associated with diagonal *couplets*—were frequently adopted at the 2h-$2^{\mathrm{nd}}$ spot, where the stair height was relatively low, compared to the 2h-$1^{\mathrm{st}}$ spot, which involved a higher staircase. Conversely, in the 2h-$1^{\mathrm{st}}$ spot, gait was characterised by [0–10]+[90–100]% diagonality region, reflecting a greater tendency toward lateral couplets. These findings suggest that walkers tended to transit between lateral and diagonal couplets patterns depending on the trail condition.

## 4 Discussion

This study examined how novice hikers adjust their gait while ascending natural terrain by using trekking poles. Importantly, the present study does not aim to explain biomechanical or stability mechanisms; rather, it focuses exclusively on classifying coordination patterns based on ground-contact timing. The findings showed that gait patterns transition flexibly in response to environmental constraints, particularly fatigue and staircase height.

Walking 1.6 km between spots significantly increased fatigue. Our MF values (≈3–5) were lower than those reported in studies linking sustained or cyclic muscle contractions to elevated RPE and lactate (18, 19), which, for example, Vargas-Molina et al. (19) reported RPE values and blood lactate levels of approximately 8 and 5–6 mmol/L, respectively, further reflecting that such a high level of muscular fatigue was unlikely here and that the fatigue we observed was too mild to consider muscular fatigue a major factor. RPE rose approximately from 4 to 6 (“somewhat hard” to “hard”), values corresponding to fatigue levels near the lactate threshold reported in previous studies of both untrained and trained participants (20–22). Moreover, the HR measured in the latter half of the trail (146–150 bpm) fell within the lactate threshold boundary values reported previously—130–150 bpm for untrained participants and 150–170 bpm for trained men and women (21). Nonetheless, the correlation between RPE, HR, and lactate has been demonstrated to vary with exercise conditions such as trail gradient (23); caution is warranted, as the RPE-lactate relationship may vary with exercise intensity and duration (24). Thus, estimating absolute fatigue levels from the subjective reports of our participants should be done cautiously; the observed increase is better interpreted as a significant but relative change induced by trail walking.

Thus, although fatigue had accumulated to some extent, as long as the stair height remained ≈ 20 cm (see the panels for 1h-$1^{\mathrm{st}}$, 1h-$2^{\mathrm{nd}}$, and 2h-$2^{\mathrm{nd}}$ spots in Figure 5), diagonal couplets were predominantly used in both the early and later course spots. Diagonal couplets characterise quadrupeds with proportionally short limbs, whose anatomical constraints necessitate diagonal limb placement beneath the torso. Cartmill et al. (9) modelled this quadrupedal structure, demonstrating that its advantage lies in preventing excessive medio-lateral body sway, enhancing support polygon stability and reducing the risk of falling. Furthermore, this adjustment helps maintain the whole-body center-of-mass within the base of support formed by the fore and hind limbs, while simultaneously projecting it anterior to the hind limbs. Additionally, such a gait pattern has been shown to predominantly emerge when humans are asked to crawl on uneven terrain using a quadrupedal gait (2, 25, 26),suggesting that diagonal couplets may be an inherent coordination mode in long-torso mammals, including humans.

In contrast, when ascending relatively high (approximately 40 cm) staircases under accumulated fatigue, the walkers exhibited lateral couplets. Although kinematic and kinetic analyses were not conducted, we speculated that ipsilateral foot-pole double support may facilitate vertical elevation of the whole-body center-of-mass to the next step. Additionally, when staircase height decreased at the 2h-$2^{\mathrm{nd}}$ spot, the frequency of diagonal couplets increased relative that at the 2h-$1^{\mathrm{st}}$ spot, while lateral couplets remained prevalent (see the [40-50]+[50-60]% and [0-10]+[90-100]% regions in the lower two panels of Figure 5). Toward the end of the trail, the coordination patterns converged to either diagonal or lateral couplets, with fewer intermediate patterns.

Regarding the symmetry structure described by group theory, trot and pace are mathematically distinct, each defined by a different symmetry organisation. Nonetheless, these are structurally related gait patterns, as one can be transformed into the other through *walking*, a specific reordering of limb coordination (27, 28). In group-theoretic terms, this relationship is known as *conjugacy*, with the walk gait functioning as a transformation that bridges these two patterns. Consider various quadrupedal gaits as elements of a subgroup Γ of the symmetry group $S_{4}$. Let the left hind leg be 1, the right hind leg be 2, the left front leg be 3, and the right front leg be 4. Among these gaits, the lateral couplets (1,3)(2,4)∈Γ and the diagonal couplets (1,4)(2,3)∈Γ represent the symmetries of pace and trot, respectively. These two patterns are mutually conjugate, and the permutation (1,3,2,4)∈Γ, corresponding to the *walk* gait, serves as the conjugating element that transforms one symmetry into the other. Nonetheless, unlike trot and pace, the bounce (1,2)(3,4)∈Γ is not conjugate to either (29). This suggests that trot and pace are special gaits that animals transition between under internal or external constraints. Such transitions may be implemented by an eight-element neural circuit $\Gamma =Z_{4}(\kappa )\times Z_{2}(\omega )$ composed of the product of a four-element cyclic group $Z_{4}(\kappa )$, involving fore-hind coordination with two hidden elements each, and a 2-element cyclic group $Z_{2}(\omega )$, governing left-right coordination (27, 28, 30). Therefore, trot and pace—that is, diagonal and lateral couplets—can be understood as gaits that continuously transition within a system represented by a single parameter, *diagonality*, which quantifies the temporal coordination between limb pairs.

To elucidate the mechanisms underlying the gait transitions identified in this study, future research should investigate the mechanical advantages of diagonal and lateral couplets under controlled laboratory conditions. For example, examining whether the transitions observed here exhibit load-dependent properties [e.g., (31) would help clarify the functional benefits associated with each coordination pattern. Advancing our understanding of these mechanisms will provide a strong basis for evaluating the convenience and safety of pole-assisted locomotion from both biological and mechanical perspectives.

A key limitation of the present study is that no kinetic, kinematic, or center-of-mass measurements were collected; therefore, our interpretations are restricted to timing-based coordination structures and do not directly address the underlying mechanical mechanisms.

## Data Availability

The raw data supporting the conclusions of this article will be made available by the authors, without undue reservation.
