# Supplementary material for: Adaptive gait transition in trekking pole-assisted hiking due to fatigue and staircase height elevation
Source: Front Sports Act Living. 2026 Jan 23;7:1669574. doi: 10.3389/fspor.2025.1669574 (PMC12876130; doi:10.3389/fspor.2025.1669574)
Supplement: Supplementary file 1 [file Datasheet1.pdf]

Supplementary Material

1 SUPPLEMENTARY DATA

We examined whether two types of patterns—sequence (lateral vs. diagonal) and couplet (lateral vs. diagonal)—could be estimated based on the diagonality measure, independent of the temporal offset between the left and right pole placements. To test this, we systematically varied the lag between the ground contact times of the two poles from 10% to 90% of one gait cycle in 10% increments. In these simulations, the duty factor was set at 48.3% for the poles and 59.3% for the feet, with the lag between the left- and right-foot contacts fixed at 50% of the gait cycle. For each of the nine resulting conditions, we generated gait diagrams (Figure 2B in the main text; not shown here). Based on these diagrams, we computed the proportion of time within a single gait cycle corresponding to lateral bipedality and diagonal bipedality, respectively, as a function of diagonality—defined as the delay in right pole contact relative to right foot contact, expressed as a percentage of the gait cycle. The results for each condition are presented in the nine panels of Figure S1.

Even when the lag between the left and right poles varies from 10% to 90%, the frequencies of unilateral and diagonal bipedality fluctuate symmetrically and in opposite phases. Furthermore, in the range in which the inter-limb lag exceeds 20%, the relative frequencies of unilateral and diagonal bipedality remain constant. Lateral bipedality is more frequent when diagonality approaches 0 or 100%, whereas diagonal coupling is more frequent when diagonality is close to 50%.

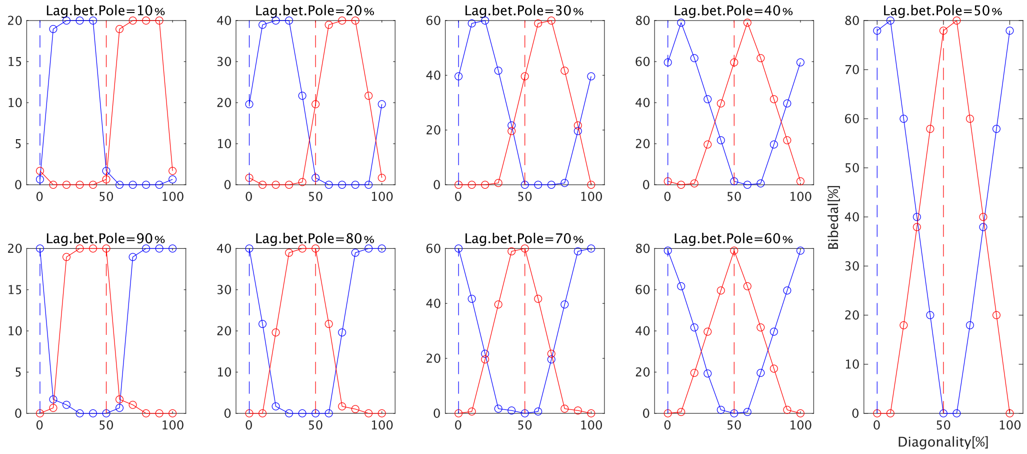

Figure S1. Percentages of unilateral (solid blue line) and diagonal (solid red line) bipedal phases as a function of diagonality in nine distinct walking patterns characterized by varying left-right pole landing time differences. A constant 50% phase offset between the legs was assumed for all patterns, except for the case in which both poles contact the ground with a lag of less than 10% of a gait cycle.
